# In-cell NMR reveals the first direct observation of endogenous interaction between HIV Tat protein and Tat RNA aptamer in human cells

**DOI:** 10.1038/s41598-025-12791-0

**Published:** 2025-08-11

**Authors:** Omar Eladl

**Affiliations:** Faculty of Pharmacy, Zagazig University, Zagazig, 44519 Egypt

**Keywords:** RNA–protein interaction, HIV Tat protein, RNA aptamer, In-cell NMR, Confocal microscopy, Nuclear localization, NOE measurements, HeLa cells, Biochemistry, Biological techniques, Biophysics, Molecular biology, Structural biology

## Abstract

RNA–protein interactions lie at the basis of numerous regulatory and functional cellular biological processes, including transcriptional control, RNA processing, nuclear export, and viral replication. Despite their fundamental biological significance, direct structural investigation of RNA–protein complexes in live human cells remains an unresolved problem due to resolution limits in spatial information, delivery of molecules, and real-time monitoring under native conditions. Existing studies rely on pre-existing in vitro complexes added to cells and therefore overlook important aspects of endogenous binding and localization. Here, we report the first in-cell nuclear magnetic resonance (NMR) study of the de novo formation of an RNA–protein complex in living human cells. By using a model system involving the HIV-1 Tat protein and its high-affinity RNA aptamer, we expressed Tat endogenously in HeLa cells and introduced the aptamer by electroporation. Direct observation was made of native complex formation within the intracellular milieu. In-cell NMR spectra exhibited characteristic chemical shift perturbations and nuclear Overhauser effect (NOE) signatures indicative of specific RNA–protein binding under physiological conditions. Comparison directly with in vitro spectra confirmed structural integrity and binding specificity in the intracellular environment. Remarkably, we obtained a partial NOE-based assignment of the RNA upon complexation with Tat in living cells—an unprecedented step towards cellular structural biology. Complementary confocal microscopy validated nuclear co-localization, enabling functionally relevant interaction. This work shows the first direct, real-time evidence for native RNA–protein complex assembly in human cells. It provides a new paradigm for probing RNA-mediated regulatory events in vivo and expands the horizon of therapeutic RNA design.

**Supplementary Information:**

The online version contains supplementary material available at 10.1038/s41598-025-12791-0.

## Introduction

The intricate RNA–protein interaction network controls a wide variety of core biological processes^1^, including mRNA splicing^2^, RNA export^3^, translation regulation^4^, and transcript stability^5^. These interactions govern the fate and function of RNA transcripts and play a pivotal role in coordinating complex cellular responses^6,7^, including those involved in cancer progression^8^, and viral infections^9,10^. Notably, viruses use highly specific RNA–protein interactions to hijack host cellular machinery for survival and replication^11^.

**Fig. 1:**
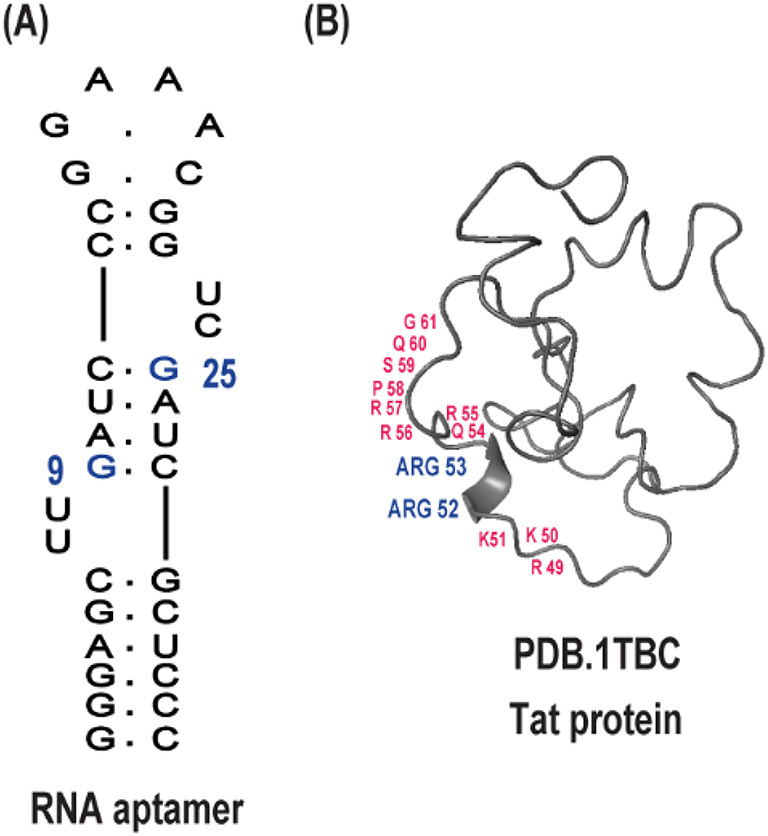
Schematic representation of HIV Tat RNA aptamer and Tat protein interaction sites. (**A**) Secondary structure model of the 34-nucleotide HIV Tat RNA aptamer. The blue highlight denotes nucleotides identified as critical for interaction with the Tat protein. (**B**) Structural representation of the full-length HIV-1 Tat protein. Amino acid residues previously reported to mediate binding to the RNA aptamer are highlighted in blue. Key residues within the basic domain, primarily responsible for RNA binding, are indicated in red.

A prominent example of such an interaction is that between HIV-1 Tat protein (14.3 kDa), an 86 amino acid (aa) intrinsically disordered protein, and the TAR (Trans-Activation Response) RNA element, located at the 5′ end of nascent viral transcripts^12–14^. Tat interacts with TAR through the arginine-rich motif (ARM), residues 49–61 (Fig. 1), and facilitates recruitment of host transcriptional elongation factors, increasing viral gene expression and replication efficiency. This interaction has been extensively characterized and is a model system for studying RNA–protein recognition and its therapeutic targeting^15^. However, most of the previous research has been conducted on in vitro systems using purified RNA and ligand components under simplified conditions^16^. Even though these experiments are valuable for gaining structural and biochemical data, they cannot accurately mimic the complexity of the intracellularenvironment, in which molecular crowding, ionic strength, and competing interactions can heavily influence binding affinity and conformational dynamics^17,18^.

One of the biggest challenges in structural biology has been observing biomolecular interactions directly within living cells^19,20^, to see how such complexes operate under physiological conditions. In-cell nuclear magnetic resonance (NMR) spectroscopy has emerged as an effective tool in studying molecular structure and dynamics within cells at atomic resolution^21–23^. While in-cell NMR has been successfully applied to small-molecule^24,25^, proteins^20,26^, and, more recently, to RNA molecules^27,28^, its application to studying RNA–protein interactions in human cells is comparatively underdeveloped. The primary obstacle lies in achieving co-delivery of RNA and protein subunits into cells at biologically relevant concentrations and molecular sizes, while maintaining their native functionality and ensuring physiological interaction.

Alternatively, early in-cell NMR studies of RNA complexes made use of pre-formed RNA–ligand or RNA–peptide complexes^27,28^. Such an approach bypasses intracellular complex assembly but does not replicate the impact of the cellular environment on interaction kinetics. Moreover, due to limitations in cell transfection size, full-length proteins are typically excluded, and short peptides or low-molecular-weight ligands are used instead^27–29^. In addition, the crowded intracellular environment and generally low affinities between partners also preclude spontaneous complex formation inside cells. Therefore, in-cell NMR studies of RNA interactions so far have relied on pre-formed complexes, limiting the biological relevance of the observations.

We report an in-cell NMR approach for real-time monitoring of RNA–protein complex assembly in real time inside living human cells. Leveraging the advantage of the high-affinity binding of the HIV-1 Tat protein with its corresponding RNA aptamer (Fig. 1). Such an aptamer was specifically engineered by SELEX to form a stable stem-loop structure that mimics the native TAR element, thus enabling high-affinity and selective recognition of the arginine-rich motif of the Tat protein, both in vitro and within cells. As we recently described^27,28,30^.

Herein, we transfected HeLa cells to intracellularly produce the Tat protein while electroporating the RNA aptamer. This two-step procedure allows for natural complex formation within the cellular environment, providing a more realistic system in which to study biologically relevant RNA–protein complexes.

Our strategy enables the first in-cell NMR detection of a full RNA–protein interaction, representing a major advance over prior studies restricted to RNA–peptide complexes. Furthermore, it allows, for the first time, real-time and endogenous monitoring of RNA interaction dynamics within living human cells. This is evidenced by the in-cell NMR spectrum, which initially displays the RNA in its free form and gradually shifts as it forms a complex with the Tat protein. This direct correspondence to complexes formed endogenously within cells and those formed in vitro brings new insight into the process of complex formation. In-cell NMR spectra exhibit chemical shift perturbations and new signal appearances consistent with specific and stable Tat binding to the aptamer. This is also indicated by in vitro NMR controls and comparative spectral analysis. Partial structural assignment of the RNA within the protein-bound form reveals conformational signatures maintaining major structural motifs. Notably, this study reports the first nuclear Overhauser effect (NOE) measurements of an RNA–protein complex in living human cells. The NOE patterns are extremely close to those that can be achieved in vitro, and this suggests that the aptamer retains its native binding conformation with the Tat protein in the cellular environment. This physiologically stable structure validates the biological relevance of the interaction and suggests the therapeutic potential of the RNA aptamer as a stable and specific inhibitor of the HIV Tat protein.

To complement our NMR findings, we employed confocal fluorescence microscopy to ascertain the intracellular distribution of the RNA and Tat protein. Co-localization of the two components within the nucleus confirms that the interaction is in the right subcellular environment and accounts for the functional relevance of the binding process.

Combined, our studies establish a new benchmark for the study of RNA–protein interactions in the direct environment of living cells using in-cell NMR. By enabling the observation of native complex assembly and structure dynamics within a biologically competent environment, this study establishes a hitherto missing piece of structural RNA biology knowledge and sets the stage for the rational design of therapeutically active RNA-targeting therapeutics in vivo.

## Materials and methods

### Cell culture and expression of HIV Tat protein in HeLa cells

HeLa cells (ATCC^®^ CCL-2™), a human cervical epithelial carcinoma cell line, were grown in high-glucose Dulbecco’s Modified Eagle Medium (DMEM) containing 10% heat-inactivated fetal bovine serum (FBS), 1% penicillin-streptomycin, and 2 mM L-glutamine. Cultures were maintained at 37 °C in a 5% CO₂ humidified atmosphere and were passed every 2 days when they became approximately 80% confluent with 0.05% trypsin-EDTA.

To express the HIV-1 Tat protein intracellularly, cells were seeded in 6-well plates at a density of 3 × 10⁵ cells/well and cultured to ~ 70% confluency overnight. Transfection was performed with 2.5 µg of a plasmid encoding the full-length Tat protein (86 amino acids), _1_MDPVDPNIEPWNHPGSQPKTACNRCHCKKCC YHCQVCFIKKGLGISYGRKKRRQRRRPSQGGQTH QDPIPKQPSSQPRGDPTGPKE_85_, under the control of a cytomegalovirus (CMV) immediate-early promoter. The Tat coding sequence was cloned into a mammalian expression vector (pcDNA3.1, Invitrogen). No GFP fusion or additional reporter elements were included in the construct to preserve native Tat functionality. Briefly, DNA was diluted in Opti-MEM and mixed with P3000 reagent, then blended with a Lipofectamine 3000 dilution and left to incubate to form complexes. The Cocktail was added dropwise to cells in antibiotic-free DMEM. Medium was replaced with complete DMEM after 6 h, and cells were incubated for an additional 48 h before analysis.

To monitor transfection efficiency, a plasmid encoding GFP was co-transfected in parallel as an internal reporter control. Importantly, GFP was not fused to the Tat protein but delivered on a separate construct to avoid interference with Tat localization or function. Protein expression was validated by Western blotting using an anti-HA monoclonal antibody (Cell Signaling Technology) and an HRP-conjugated secondary antibody (Abcam). Detection was performed using enhanced chemiluminescence (Thermo Scientific).

### HIV Tat protein expression in *E. coli*

To express the HIV-1 Tat protein in *E. coli*, the full-length Tat protein was cloned into thebacterial expression vector pET-28a (+), which has a His6 tag for purification. The vector was transferred into the BL21(DE3) strain of *E. coli*, and protein expression was induced by adding 1 mM isopropyl-β-D-thiogalactopyranoside (IPTG) to the culture when the optical density (OD600) was 0.6. The culture was incubated at 18 °C for 16 h with shaking at 200 rpm. The cells were then harvested by centrifugation and resuspended in a lysis buffer (50 mM Tris-HCl, 150 mM NaCl, 1 mM EDTA, 1 mM DTT, pH 8.0). The cell suspension was sonicated, and the lysate was centrifuged to clear cell debris.

The Tat protein was initially purified by Ni-NTA affinity chromatography (Qiagen) under denaturing conditions. The soluble fraction was loaded on a pre-equilibrated Ni-NTA agarose column, and the bound protein was washed using a wash buffer (50 mM Tris-HCl, 150 mM NaCl, 20 mM imidazole, 6 M guanidine-HCl, pH 8.0). The protein was eluted with an elution buffer (50 mM Tris-HCl, 150 mM NaCl, 250 mM imidazole, 6 M guanidine-HCl, pH 8.0). The Eluted protein was dialyzed against a buffer (20 mM Tris-HCl, 150 mM NaCl, 1 mM DTT, pH 7.5) for the purpose of denaturant removal and protein refolding.

For high purity, the protein was further purified by Fast Protein Liquid Chromatography (FPLC). The protein sample was loaded onto a Superdex 75 h 10/30 gel filtration column (GE Healthcare) pre-equilibrated with buffer A (20 mM Tris-HCl, 150 mM NaCl, pH 7.5). The protein was eluted using a linear gradient of buffer B (20 mM Tris-HCl, 150 mM NaCl, 1 M urea, pH 7.5) from 0 to 100%. Fractions containing the target Tat protein were pooled and dialyzed with buffer C (20 mM Tris-HCl, 150 mM NaCl, 1 mM DTT, pH 7.5) to both remove urea and refold the protein.

The protein was concentrated using an Amicon centrifugal filter unit (Millipore) with a 10 kDa molecular weight cutoff. The concentration of the purified protein was determined using the Bradford assay, and purity was verified using SDS-PAGE. The protein was stored at -80 °C for subsequent use.

### Preparation of RNA Tat aptamer

The sequence of the Tat aptamer was derived from previously characterized RNA high-affinity binders (5′-GGGAGCUUGAUCCCGGAAACGGUCGAUCGCUCCC-3′). A DNA template for the RNA sequence was created by PCR amplification using T7 promoter-containing primers. The PCR product was cleaned using a QIAquick PCR Purification Kit (Qiagen).

In vitro transcription was performed using a HiScribe T7 High Yield RNA Synthesis Kit (New England Biolabs). The transcription reaction (total volume 100 µL) contained 1 µg DNA template, 7.5 mM each NTP, 10× T7 buffer, and 2 µL of T7 RNA polymerase. The reaction was incubated at 37 °C for 4 h. Following transcription, the reaction was treated with DNase I (New England Biolabs) to digest the DNA template.

RNA was subsequently purified by 10% denaturing urea–polyacrylamide gel electrophoresis (urea-PAGE) in a 1× TBE buffer. The gel was visualized by UV shadowing, the appropriate band excised and eluted in elution buffer (300 mM NaOAc, pH 5.2, 1 mM EDTA, 0.1% SDS) overnight at 4 °C. RNA was ethanol precipitated, washed in 70% ethanol, and resuspended in nuclease-free water. Concentration was determined spectrophotometrically (Nanodrop) using A₂₆₀.

For NMR use, RNA was annealed by heating at 95 °C for 3 min in annealing buffer (5 mM MgCl, 10 mM sodium phosphate, pH 6.5) and gradually cooled to room temperature over 1 h. For HSQC and NOESY measurement and Confocal Microscopy, the labeled RNA was synthesized, purified, and desalted by Integrated DNA Technologies (IDT, Coralville, IA, USA).

### NMR titration of Tat protein into RNA aptamer

In vitro NMR titration experiments were conducted to study the interaction between the HIV-1 Tat protein and its RNA aptamer. All measurements were made on a 600 MHz Bruker Avance III spectrometer equipped with a cryoprobe at 20 °C. The RNA aptamer was dissolved to a final concentration of 100 µM in NMR buffer consisting of 10 mM sodium phosphate, 5 mM MgCl, and 0.1 mM EDTA (pH 6.5), with an addition of 10% D₂O for field locking.

Tat protein was titrated incrementally into the RNA sample to a final RNA: Tat molar ratio of 1:0, 1:0.25, 1:0.5, 1:0.75, 1:1, 1:1.5, and 1:2. After each addition, samples were gently mixed and allowed to equilibrate for 10–15 min prior to acquisition.

1D 1 H NMR spectra were measured after each titration step, monitoring the region of the imino protons (10–15 ppm). Chemical shift changes and the appearance of new peaks were assessed to establish the formation and degree of RNA-protein complex formation. Processing and analysis of the spectra were performed using TopSpin (Bruker).

### Electroporation of RNA into HeLa cells

Electroporation was performed using the Gene Pulser Xcell Electroporation System (Bio-Rad) in 0.4 cm cuvettes. HeLa cells were harvested 48 h post-transfection, washed twice with PBS, and resuspended in electroporation buffer (CytoPoration Medium T, BTX Harvard Apparatus) at a concentration of 1 × 10⁷ cells/mL. For each electroporation, 400 µL of cell suspension was mixed with 1 mM of refolded RNA aptamer and placed in an ice-chilled cuvette.

Electroporation was carried out at 250 V and 950 µF with an infinite resistance setting. Cells were transferred immediately to pre-warmed complete DMEM following the pulse and incubated for 1 h at 37 °C to recover before washing in the NMR buffer.

### In-cell NMR spectroscopy

Cells were washed twice in PBS and NMR buffer containing 10 mM sodium phosphate (pH 6.5), 5 mM MgCl, and 10% D₂O for field lock, then resuspended at 1 × 10⁸ cells/mL. Cell viability was > 90% as assessed by trypan blue exclusion.

To ensure that no extracellular RNA or protein remained after transfection, the final NMR buffer supernatant was collected following the washing steps and analyzed by 1D¹H NMR (imino region), confirming the absence of signal and validating an intracellular origin.

In-cell NMR spectra were acquired using a Bruker Avance III HD 600 MHz spectrometer equipped with a 5 mm TXI cryoprobe. The samples were positioned in Shigemi tubes and maintained at 20 °C inside a bioreactor system (Bruker InsightCell™) with constant perfusion of oxygenated medium30,31. A 1D¹H standard spectrum was recorded at 1, 2,3, 4, 6, 8, and 12 h after electroporation to monitor complex formation.

The bioreactor system maintained media exchange at a flow rate of 50 µL/min, which preserved cell viability and actively flushed out any potential leakage products during extended acquisition. A post-experiment 1D¹H spectrum of the supernatant was also acquired and showed no signal, further confirming the intracellular origin of the observed NMR resonances.

Data was processed using TopSpin (Bruker) and analyzed with Poky. Chemical shift changes were compared with reference in vitro spectra under identical buffer and temperature conditions.

### In-cell HSQC and NOESY NMR measurements

To investigate the molecular details of the HIV Tat protein interaction with the ¹⁵N–¹³C labeled RNA aptamer inside living cells, we first acquired a two-dimensional ¹H–¹⁵N HSQC spectrum. This experiment enabled us to assess RNA folding and base pairing integrity under in-cell conditions. The HSQC spectrum was recorded at 20 °C on a Bruker 600 MHz spectrometer equipped with a cryoprobe, using 256 scans, with spectral widths of 12 ppm (¹H) and 30 ppm (¹⁵N) centered at ~ 8.5 ppm and 150 ppm, respectively. The HSQC signals served as a reference for secondary structure elements and were used to guide the assignment of imino resonances in subsequent experiments.

To further confirm the interaction of the HIV Tat protein with the labeled RNA aptamer and extend the use of in-cell NMR methodologies even further, we performed the first ever in-cell NOESY measurement. This involved the quantitation of ¹⁵N–¹³C NOE cross-peaks in living HeLa cells that were imbued with the RNA aptamer and Tat protein. The NOESY experiment was acquired using the same Bruker 600 MHz NMR spectrometer with a cryoprobe, with the average acquisition time being 12 h. The NOESY experiment was conducted at 20 °C with a 300 ms mixing time. 256 scans were collected, with an acquisition time of 1.2 s per scan. The data were processed with standard Bruker software, and the chemical shifts were assigned manually and automatically using Poky software.

### Confocal fluorescence microscopy

HeLa cells were seeded on poly-D-lysine–coated coverslips and incubated overnight. First, Cells were electroporated with TAMRA-labeled Tat protein expression. After 48 h of expression, FAM-labeled RNA was electroporated using a two-porating pulse and five transfer pulses. Electroporated cells were placed back in the incubator for recovery.

One-hour post-electroporation, cells were washed with PBS and fixed in 4% paraformaldehyde for 15 min at room temperature. Nuclei were stained with Hoechst dye, and coverslips were mounted in antifade reagent. Images were acquired on a Zeiss LSM 880 microscope with Airyscan at 63× magnification. Intracellular fluorescence was visualized in the green (FAM) and violet (TAMRA) channels.

## Results

### Expression of HIV Tat protein in HeLa cells and *E. coli*

**Fig. 2:**
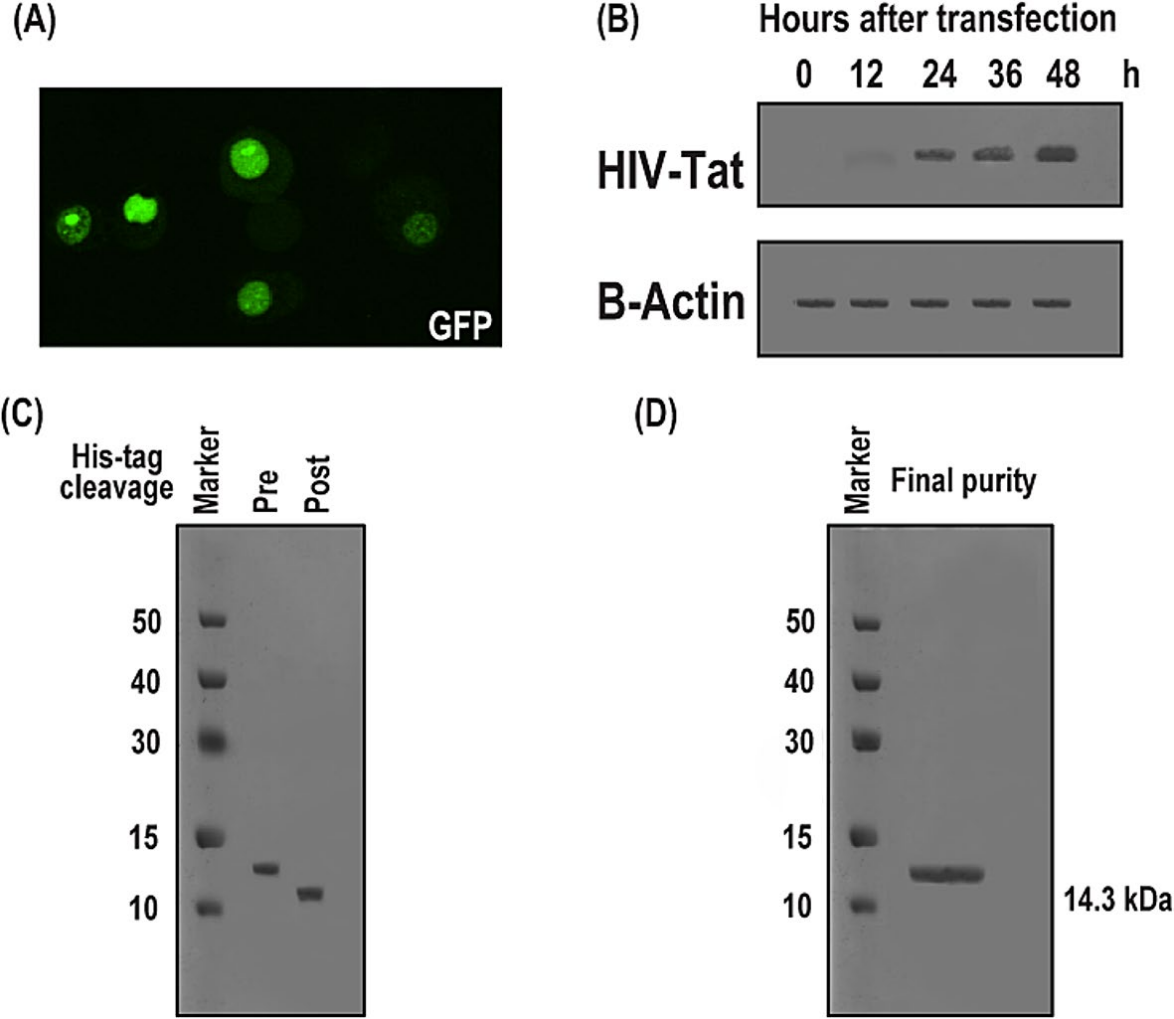
Expression and purification of HIV-1 Tat protein in HeLa cells and *E. coli*. (**A**) Fluorescence microscopy of HeLa cells co-transfected with a GFP-expressing reporter plasmid, confirming transfection efficiency through GFP signal visualization. (**B**) Time-course Western blot analysis of Tat expression in HeLa cells from 0 to 48 h post-transfection. A distinct band corresponding to the expected molecular weight of Tat (14.3 kDa) is observed, with β-actin used as a loading control. (**C**) Expression of recombinant full-length HIV-1 Tat protein in E. coli BL21(DE3). The protein was expressed with an N-terminal His₆ tag, which was subsequently cleaved after purification. SDS-PAGE analysis shows robust expression in the soluble fraction. (**D**) Final purity of the Tat protein following Ni-NTA affinity chromatography and size-exclusion chromatography. A single major band on SDS-PAGE confirms high purity suitable for downstream applications. The full-length uncropped gels are provided in the Supplementary Figure 1.

To set up theintracellular expression of full-length HIV-1 Tat for both in-cell and in vitro interaction studies, we first confirmed its expression in HeLa cells (Fig. 2 and Supplementary Fig. 1). Transfection efficiency was assessed using a co-transfected GFP reporter plasmid. Green fluorescence confirmed successful transfection and GFP expression (Fig. 2A), and served as a marker indicating that GFP-positive cells were co-transfected with Tat.

Western blotting performed 48 h post-transfection showed a clear band at the expected 14.3 kDa molecular weight, confirming robust Tat expression (Fig. 2B). β-actin served as a loading control to normalize Tat levels across time points (0, 12, 24, 36, 48 h), with expression peaking at 48 h.

To obtain sufficient Tat protein for in vitro NMR studies, the same construct was also expressed in *E. coli* BL21(DE3) (Fig. 2C,D). Expression at low temperature improved the solubility of the His₆-tagged Tat protein. SDS-PAGE analysis confirmed high-level expression (Fig. 2C). The protein was purified via denaturing Ni-NTA affinity chromatography, followed by on-column refolding and final polishing using size-exclusion chromatography (FPLC, Superdex 75). The final product was pure, with a single-peak elution profile and clean SDS-PAGE band (Fig. 2D), suitable for in vitro and in-cell NMR experiments.

### RNA-protein complex formation: (in vitro NMR)

To investigate the binding of the HIV-1 Tat protein to its target RNA aptamer, we conducted a series of in vitro NMR titrations focusing on the imino proton region, which selectively reports on RNA hydrogen-bonded base pairs and excludes protein signals. Incremental additions of Tat protein were made to a fixed concentration of RNA aptamer, reaching a final RNA: Tat molar ratio of 1:2.

**Fig. 3:**
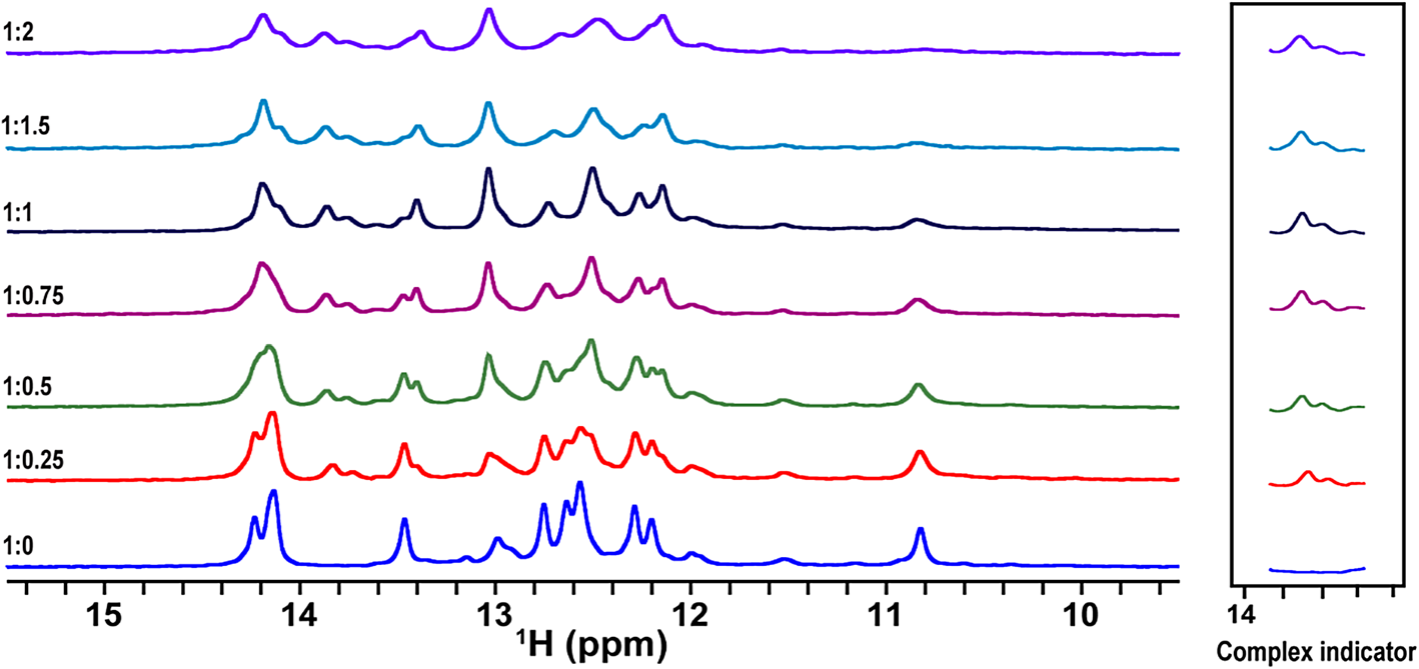
In vitro titration of HIV-1 Tat protein into Tat RNA aptamer monitored by 1D Imino proton NMR. ^1^H NMR spectra were recorded during the titration of purified HIV-1 Tat protein into the Tat RNA aptamer at 20 °C. Each spectrum corresponds to an incremental concentration of Tat protein, revealing progressive chemical shift perturbations indicative of complex formation. The boxed region highlights specific imino proton signals that undergo notable changes upon binding, confirming direct interaction between the aptamer and Tat protein.

Upon each Tat addition, stepwise chemical shift perturbations and the appearance of new peaks (13.7 and 13.8 ppm) in the imino region were observed as indicators of the process of complex formation (Fig. 3). Under a slow exchange regime, these included a distinct two-signal pattern associated with UAU base triple formation assigned as U7 and U23, alongside broader perturbations reflecting structural rearrangements of the RNA upon binding, consistent with what was previously described^28^. These spectral changes were absent in spectra of free RNA and were concentration-dependent, which guaranteed specific binding between the Tat protein and the aptamer. Most importantly, there was saturation in binding at about the 1:1 ratio, with no additional change in the spectrum after further addition of Tat, suggesting the formation of a well-characterized and stoichiometric RNA–protein complex.

This titration experiment series clearly shows that the HIV-1 Tat protein is directly bound by the RNA aptamer, forming a stable complex that induces characteristic and reproducible alterations of the hydrogen-bonded base pairs of the RNA, as seen in the imino spectral region (Fig. 3).

### In-cell NMR and structural insights

Upon in vitro validation, the RNA aptamer-Tat protein interaction was further probed in the cellular context using in-cell NMR.

**Fig. 4:**
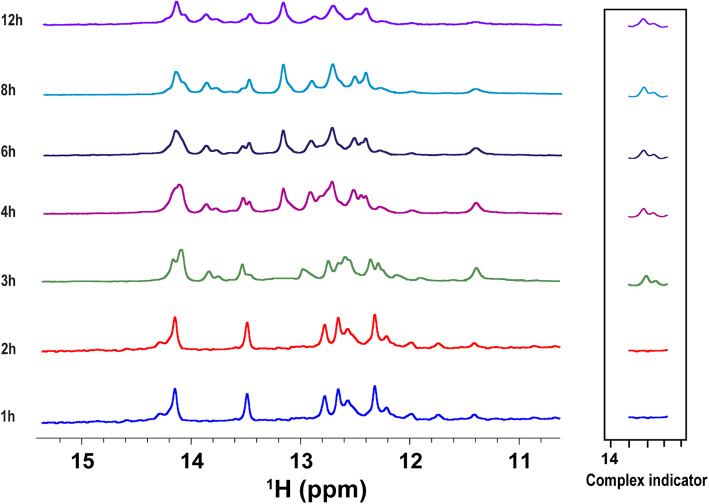
Time-resolved in-cell NMR of Tat RNA aptamer in HeLa cells overexpressing HIV-1 Tat protein. ^1^H NMR imino spectra recorded from HeLa cells transfected with the Tat RNA aptamer and co-expressing HIV-1 Tat protein. Spectra were acquired at various time points post-transfection (0, 2, 3, 4, 6, 8, 12 h) to monitor the intracellular interaction and complex formation. Specific imino proton signals, highlighted in the boxed region, exhibit time-dependent changes consistent with the RNA–protein complex formation inside live cells at 20 °C.

HeLa cells expressing Tat protein were transfected with the RNA aptamer (Fig. 4). In the first hour of in-cell NMR measurement, the imino spectrum indicated signals corresponding to the free RNA aptamer without any observable changes. Starting from the second hour, new signals began to appear in the imino region (13.7 and 13.8 ppm), indicating complex formation between the RNA aptamer and Tat protein in vivo (Fig. 4). These are the same signals of U7 and U23 identified as indicators of RNA–protein complex formation during in vitro titration (Fig. 3). In consistency with our previous publication^28^the new signals observed were absent in free RNA spectra, thereby confirming that the Tat protein had bound to the RNA aptamer in vivo. Additionally, control spectra of the extracellular medium before and after in-cell NMR measurements showed no detectable RNA signals, confirming the intracellular origin of the observed resonances (Supplementary Fig. S2).

### In-cell NMR HSQC and NOE measurements

**Fig. 5:**
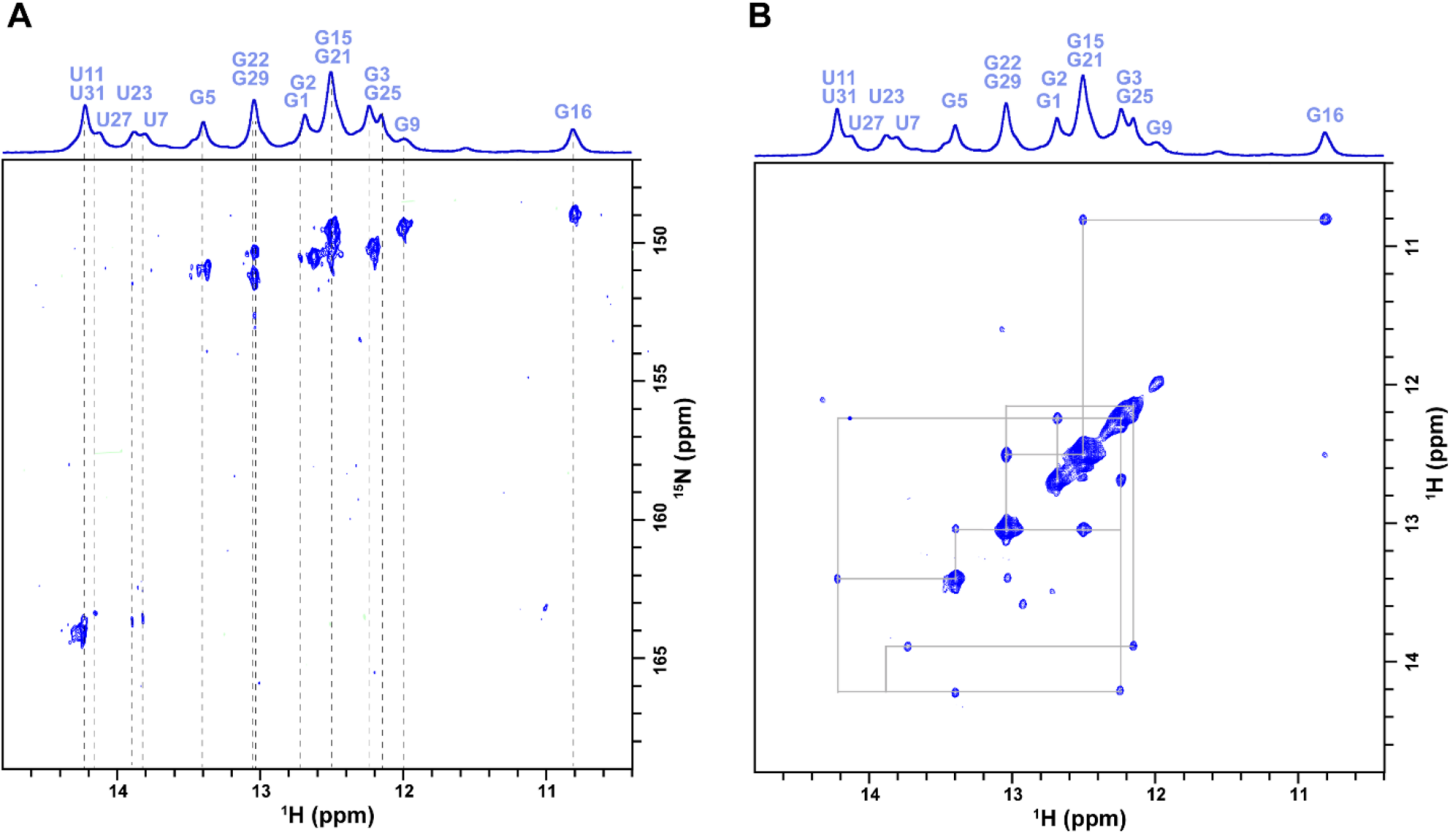
In-cell NMR of Tat RNA aptamer in complex with HIV-1 Tat protein. (**A**) Two-dimensional ¹H–¹⁵N HSQC spectrum acquired from HeLa cells expressing HIV-1 Tat protein and transfected with uniformly ¹⁵N-labeled Tat RNA aptamer. The appearance of imino cross-peaks confirms the intracellular folding and stabilization of the aptamer in the cellular environment. (**B**) Two-dimensional ¹H–¹H NOESY spectrum recorded under the same conditions reveals distinct cross-peaks corresponding to short-range spatial contacts within the RNA aptamer and at the RNA–protein interface. The presence of intra- and intermolecular NOEs confirms stable complex formation in the intracellular milieu. All spectra were acquired at 20 °C .

To acquire detailed structural information for the interaction interface of HIV-1 Tat protein and RNA aptamer under native cell conditions, we first recorded in-cell two-dimensional ¹H–¹⁵N HSQC spectra using uniformly ¹³C-¹⁵N-labeled RNA aptamer (Fig. 5A). The HSQC spectrum revealed well-dispersed imino signals from the RNA within the live HeLa cells, indicating that the aptamer retained a defined secondary structure in the complex intracellular environment. Notably, peaks corresponding to U7 and U23 involved in UAU base triple formation at 13.7 and 13.8 ppm, respectively, were observed, representing key residues involved in Tat binding and confirming the complex formation in vivo.

We then conducted Nuclear Overhauser Effect (NOE)measurements using in-cell NMR spectroscopy (Fig. 5B). Isotopic labeling enabled us to obtain high-resolution NOESY spectra directly from living cells. The in-cell NOESY spectra revealed several inter- and intramolecular NOE cross-peaks between the RNA aptamer and Tat protein. Among these, NOE signals involving U7 and U23 were identified, consistent with their spatial proximity to amino acid side chains on the binding interface. Comparison with our previously published NOE results in vitro, obtained from the RNA–Tat complex in buffer, demonstrated a very good concordance in NOE patterns, reflecting that the binding conformation of the complex is mostly preserved within the cell^28^.

Despite the in-cell NOE cross-peaks usually being weaker, especially for non-conical base triplets like UAU and broader than their in vitro counterparts due to cellular viscosity, reduced molecular tumbling, and dense background, their reproducibility and agreement with the in vitro dataset confirm their biological relevance. Of particular interest, this spectrum is the first successful 2D in-cell NOESY measurement of an RNA–protein complex and sets a precedent for the use of NOE-based NMR to define RNA-protein interfaces at atomic resolution within living cells (Fig. 5B).

### Confocal microscopy analysis of the intracellular localization and co-localization of Tat RNA aptamer and HIV-1 Tat protein

To correlate our in-cell NMR data and to observe subcellular localization of the RNA–Tat protein complex, we used high-resolution confocal fluorescence microscopy. HeLa cells were co-transfected with a plasmid coding for the full-length HIV-1 Tat protein fused with a fluorescent reporter (TAMRA). After 48 h, cells were electroporated with a FAM-conjugated RNA aptamer. After fixation, cells were counterstained using Hoechst to stain the nucleus and thereafter observed using a confocal microscope.

**Fig. 6:**
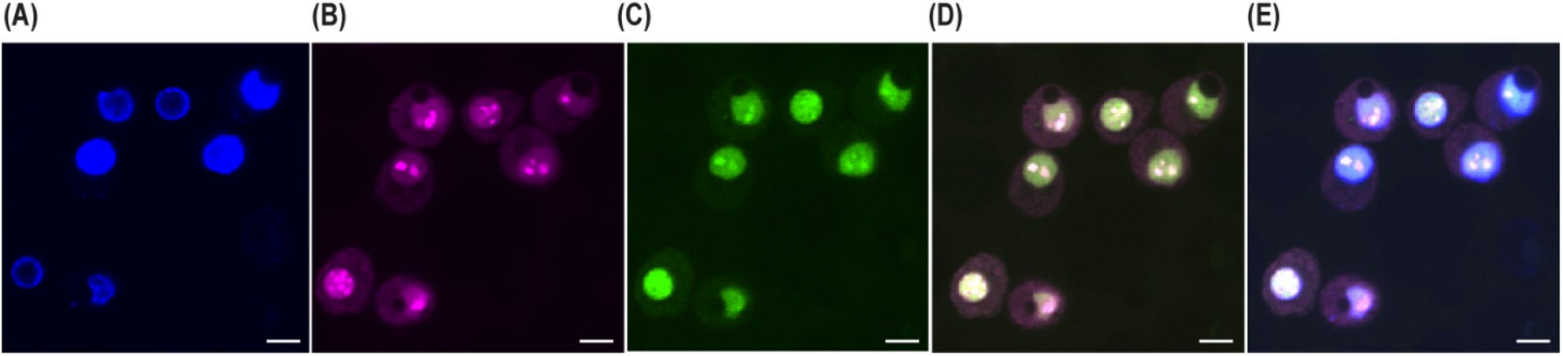
Confocal microscopy of Tat RNA aptamer and HIV-1 Tat protein in HeLa cells. HeLa cells expressing TAMRA-tagged Tat protein were electroporated with FAM-labeled RNA aptamer. Confocal images show (**A**) brightfield, (**B**) Tat protein (TAMRA), (**C**) RNA aptamer (FAM), (**D**) merged Tat and aptamer signals, and (**E**) overlay with Hoechst-stained nucleus. Scale bar = 10 μm.

Confocal imaging (Fig. 6) showed high Tat protein expression in the HeLa cells (Fig. 6A), with a predominantly nuclear pattern consistent with its well-defined role as a transcriptional activator (Fig. 6B). Notably, the fluorescent signal for the RNA aptamer (Fig. 6C) was also localized within the nucleus, with significant spatial overlap with the Tat protein signal, indicating a very high degree of co-distribution between the two molecular populations in the nucleus of HeLa cells (Fig. 6D). The merged overlay (Fig. 6E) shows co-localization of blue Hoechst-stained nucleus, violet Tat protein, and green Tat RNA aptamer.

## Discussion

The current work is the first successful in-cell NMR investigation of an RNA-protein interaction between the Tat RNA aptamer and HIV-1 Tat protein in living human cells. Intracellular expression of the Tat protein in HeLa cells and electroporation delivery of the RNA aptamer enabled us to monitor the formation and structural dynamics of the resulting RNA-protein complex in real time within the cellular environment. This approach bridges the gap between in vitro experiments of the traditional kind and the actual biological environment under which such molecular interactions occur, offering an increasingly powerful system for research into RNA-targeting strategies of immediate therapeutic relevance.

Here, our in vitro NMR titration experiments provided compelling evidence for the specific binding of HIV-1 Tat protein and its RNA aptamer. Changes in the imino proton shifts and emergence of a new signal suggested base-pairing perturbations and structural rearrangements upon formation of the complex. The emergence of new well-defined peaks in the imino region was proportional to increasing Tat protein concentrations, establishing a saturable, high-affinity interaction. These in vitro spectral signatures served as a comparative basis to validate binding within cells and confirm complex formation under physiological conditions.

In-cell NMR experiments in live HeLa cells allowed us to observe these very same binding interactions in real-time within a native biological environment. Initially, the imino spectrum reflected the conformation of the free RNA in the cells. Over time, however, we observed new imino signals that recapitulated those seen during in vitro binding. The delayed appearance of complex-specific signals reflects the endogenous nature of molecular recognition and real-time intracellular complex formation. This can be explained by several contributing factors: (1) the RNA aptamer requires time to translocate into the nucleus where Tat is localized; (2) intracellular diffusion and folding kinetics of the aptamer contribute to delayed target engagement; and (3) initial concentrations of complexed RNA may be below the NMR detection threshold due to dynamic binding or low accumulation inside the extremely crowded cellular environment. Fortunately, this confirms that the observed spectral changes are not artifacts but represent true, biologically driven interaction kinetics occurring within live cells. This real-time capture of complex formation directly inside cells reinforces the value of in-cell NMR in probing dynamic macromolecular behavior.

Further structural details were obtained from in-cell HSQC and NOESY experiments. Despite the limitations of reduced sensitivity and increased backgroundin cellular samples, we found significant HSQC and NOE cross-peaks corresponding to interfaces at the RNA-protein interface. These results were consistent with NOE patterns detected in vitro and represented direct evidence of spatial proximity between Tat protein and RNA residues in the cellular environment. This work is a significant milestone in the application of in-cell NOESY to RNA-protein systems, providing unprecedented atomic-level information regarding their in vivo interaction.

Confocal microscopy served as an ancillary technique to locate and prove the interaction. Visualization clearly showed nuclear accumulation of both the Tat protein and the RNA aptamer following electroporation and expression, with strong colocalization within the nuclear compartment. These results are in agreement with known nuclear activity of Tat in HIV transcriptional stimulation and show that the aptamer is cell-associated and properly trafficked to the correct subcellular compartment for interaction.

Cumulatively, these findings provide compelling evidence for the utility of in-cell NMR as a method in structural biology to define functional biomolecular interactions in intact human cells. Unlike traditional assays, our in-cell NMR permits real-time observation of conformational states, binding, and dynamic protein-RNA rearrangements in their native environment. Our results confirm that the RNA aptamer is a structurally stable entity within cells, selectively interacts with Tat, and appropriately localizes for functional interaction.

This platform provides access to a wide range of applications. It offers a new path toward elucidating RNA-protein interactions that regulate viral replication, gene regulation, and epigenetic control. In addition, the ability to capture accurate atomic-level information regarding RNA interactions in vivo improves the translational value of RNA-based therapeutics and diagnostics.

In the future, this method will be applicable to evaluate other RNA-binding proteins, screen chemically stabilized or mutated aptamers, and screen small molecules that disrupt pathological RNA-protein interactions. Furthermore, combining in-cell NMR, cryo-EM, or live-cell imaging techniques could enhance spatial and temporal resolution to achieve a global overview of RNA biology in health and disease.

In brief, this work sets a precedent for the structural investigation of RNA-protein complexes in human cells and underscores the strength of combining in-cell NMR with complementary biophysical and imaging techniques. The robust binding, nuclear specificity, and intact structure of the RNA aptamer described here reinforce its value as a functional Tat inhibitor and promising lead compound for the synthesis of anti-HIV therapeutics.

## Conclusion

This study presents the first successful use of in-cell NMR spectroscopy to detect and monitor an RNA–protein interaction in real time within living human cells. By expressing full-length HIV-1 Tat protein endogenously in HeLa cells and introducing its specific RNA aptamer, we achieved direct observation of complex formation under physiological conditions.

The results confirmed that the RNA aptamer remains structurally intact and selectively interacts with Tat protein in its native nuclear environment. These findings validate the aptamer’s intracellular stability and functional specificity, reinforcing its utility as a molecular probe.

This work also demonstrates the power of in-cell NMR to capture biologically relevant structural changes and dynamic interactions that cannot be fully recapitulated in vitro. Complementary evidence from NOE-based contacts and confocal microscopy further supports the intracellular RNA–Tat binding and nuclear co-localization.

Importantly, our approach establishes a valuable framework for investigating RNA–protein interactions in intact cells at atomic resolution. This can be extended to study other regulatory RNA–protein pairs and may accelerate the development of RNA-based therapeutic tools and diagnostic strategies in a cellular context.

## Electronic supplementary material

Below is the link to the electronic supplementary material.

Supplementary Material 1

## Data Availability

All data is provided within the manuscript or supplementary information files, if present; more information is available on the hyperlink as Data Availability statement page.
