# Supplementary material for: In-cell NMR reveals the first direct observation of endogenous interaction between HIV Tat protein and Tat RNA aptamer in human cells
Source: Sci Rep. 2025 Aug 11;15:29373. doi: 10.1038/s41598-025-12791-0 (PMC12339945; doi:10.1038/s41598-025-12791-0)
Supplement: Supplementary file 1 — Supplementary Material 1 [file 41598_2025_12791_MOESM1_ESM.pdf]

Title:

In-cell NMR reveals the first direct observation of endogenous interaction between HIV Tat protein and Tat RNA aptamer in human cells

Authors:

Authors:

Omar Eladl[1, 2, *]

[1]Faculty of Pharmacy, Department of Pharmaceutical Medicinal Chemistry, King Salman International University (KSIU), Rus Sudr, Egypt.

[2]Faculty of Pharmacy, Zagazig University, Zagazig 44519, Egypt

*Corresponding author

omarsobhyeladl@yahoo.com

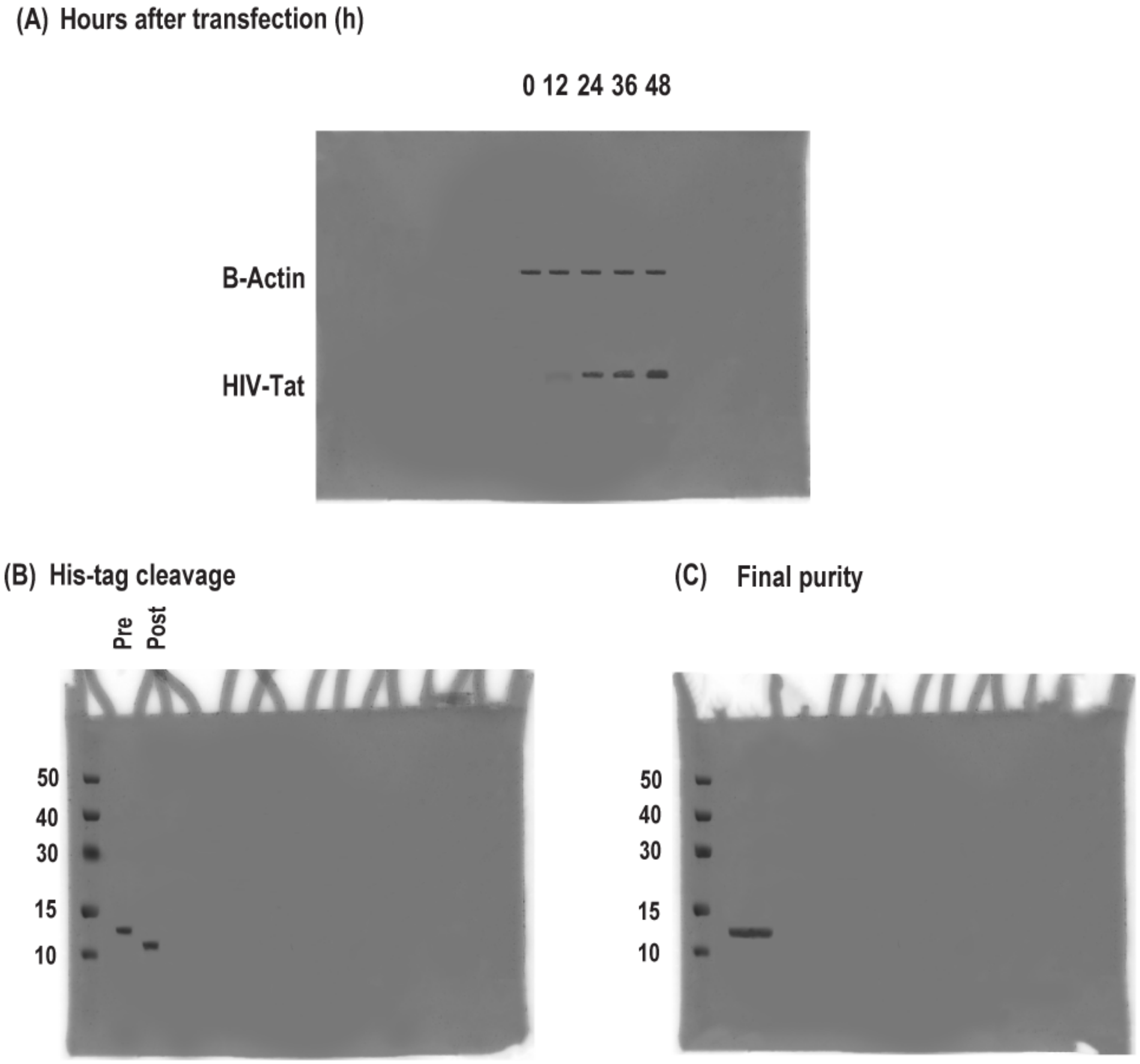

Supplementary Figure 1. Uncropped Gels for the Expression and Purification of HIV-1 Tat Protein. (A) Uncropped Western blot of Tat expression in HeLa cells, corresponding to the time-course analysis shown in Figure 2B. The full membrane image shows the entire blot, including the β-actin loading control. (B) Uncropped SDS-PAGE gel showing recombinant full-length HIV-1 Tat protein expression in E. coli BL21(DE3), corresponding to Figure 2C. The complete gel image demonstrates robust protein expression in the soluble fraction. (C) Uncropped SDS-PAGE gel of the purified Tat protein following Ni-NTA affinity chromatography and size-exclusion chromatography (Superdex 75), corresponding to Figure 2D. The full gel demonstrates the final purity and homogeneity of the Tat protein.

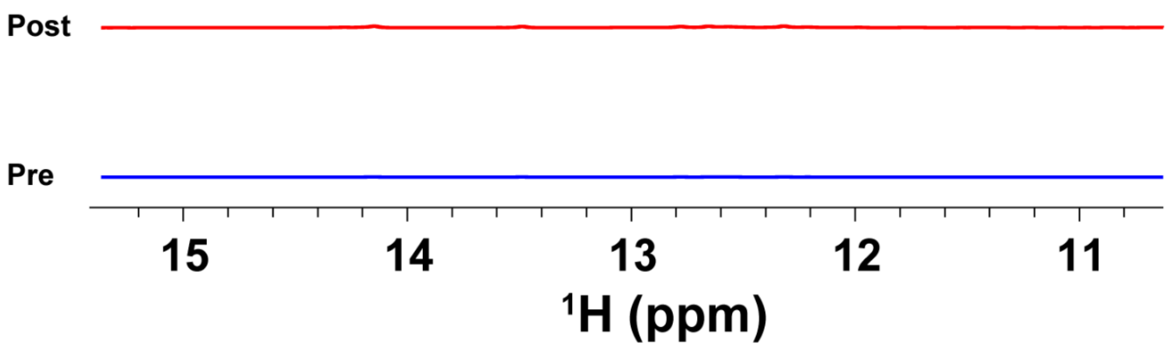

Supplementary Figure 2. 1D ^{1}H NMR spectra of the supernatant before and after in-cell NMR acquisition.
